# Direct cryopreservation of winter-acclimated buds of *Dracocephalum austriacum* (Lamiaceae) from field material

**DOI:** 10.1007/s11240-020-01850-1

**Published:** 2020-05-19

**Authors:** Thomas Rasl, Mona Schalk, Eva Temsch, Andrea Kodym

**Affiliations:** 1 Department of Pharmacognosy, University of Vienna, 1090 Vienna, Austria; 2 Department of Botany and Biodiversity Research, University of Vienna, 1030 Vienna, Austria; 3 Core Facility Botanical Garden, University of Vienna, 1030 Vienna, Austria

**Keywords:** Cold acclimation, Field material, Flow cytometry, In vitro, Micropropagation, Vitrification

## Abstract

This study develops protocols for the micropropagation and cryopreservation of *Dracocephalum austriacum* (Lamiaceae). It is a perennial herbaceous plant that overwinters with ground-level sprouts and is classified as critically endangered in Europe. In vitro cultures were initiated from seeds on growth-regulator-free Murashige & Skoog (MS) medium after nicking the seed coat. Propagation via shoot culture was achieved on ½ MS medium with 1 µM benzyl adenine (BAP). Rooting on various indole-3-butyric acid (IBA)-media was not reliable, but the rooting success was 80% after 10 weeks on medium with 1 µM BAP. Two starting materials underwent cryopreservation: (1) shoot tips from cold-acclimated in vitro plantlets and (2) axillary buds from winter shoots from field plants. For the cryopreservation of in vitro shoots, plant vitrification solution (PVS)3 and incubation over ice yielded the best results (~ 34% regeneration success). However, regeneration using winter acclimated buds were 100, 76 and 30% for collections in December, February and March, respectively, using the same protocol. Moreover, the ploidy levels of cryopreserved plantlets were estimated using flow cytometry. The use of winter-acclimated field material of temperate herbaceous plants or subshrubs has high potential as explant source for cryopreservation and calls for exploring this technique for other species.

## Key message

The novelty of this study is to use winter-acclimated buds from field material for the cryopreservation of an endangered dicotyledonous herbaceous plant.

## Introduction

Austria’s range of landscapes comprises approximately 2950 vascular plant species (Rabitsch and Essl 2008) and is host to a significant portion of threatened European species. Alongside all European Union (EU) member states, Austria has committed to the Global Strategy for Plant Conservation (GSPC), a worldwide initiative for halting biodiversity loss by advancing plant conservation. One GSPC target is to have at least 75% of the threatened plant species in ex situ collections (CBD 2019). Ex situ conservation measures include seed storage in seed banks, in vivo living plant collections, e.g. in botanical gardens, as well as in vitro culture and cryopreservation.

The Austrian dragonhead (*Dracocephalum austriacum*) belongs to the family Lamiaceae and is a perennial, herbaceous plant. The main stems die back in autumn, and winter sprouts, i.e. highly compact stems with numerous axillary buds form directly above soil surface. *D. austriacum* is recognized as endangered in Europe and listed in Appendix II and IV of the EU’s Fauna-Flora-Habitat Guidelines, which specify a strict protection regime (The Council of the European Communities 1992). Its native range spreads over Central and Eastern Europe, with outposts in the Spanish and French Pyrenees in the west to populations in Ukraine, the Caucasus and Turkey in the east. It grows mainly on dry grasslands and south-facing rocky slopes in a continental climate and prefers humus-rich soils on limestone bedrock (Käsermann 1999). The distribution of this species is very scattered and uncommon. In Austria, two small populations of *D. austriacum* have been recorded. Major threats include changes in landscape management along with over-collection because of its attractive flowers (Käsermann 1999).

The easiest and preferred way to store germplasm is in form of seeds in seed banks. However, many plants are vegetatively propagated or are large seeded or have recalcitrant (i.e. desiccation intolerant) seed. The conventional method of conserving the germplasm of these species is in living collections e.g. in orchards or botanic gardens or in in vitro collections. Living collections are also important for plants on the brink of extinction, for which ideally copies of all field plants are taken into ex-situ conservation. Cryopreservation is an important complementary tool for the preservation of plant genetic resources of crops and crop wild relatives as well as endangered species and medicinal plants (Li and Pritchard 2009; Engelmann 2011; Reed et al. 2011; Kaczmarczyk et al. 2012; Pence 2013; Matsumoto 2017; Kundu et al. 2018). Numerous methods have been developed over the years, with cryoplates being one of the latest developments (Yamamoto et al. 2011). Propagules for cryopreservation are commonly (1) shoot tips from in vitro-grown plantlets, (2) deeply dormant buds, e.g. of temperate trees or shrubs or (3) embryos and embryonic axes of large seeded or recalcitrant plants. The choice of method depends on the species.

For temperate woody plants the easiest technique is to cryopreserve dormant winter buds directly from field plants during the cold period (Towill and Ellis 2008). For field plants, freezing tolerance is naturally induced by cold-temperature exposure and short day length. The direct isolation of cold-acclimated buds is a simple technology, and recovery does not always involve in vitro culture but can also be achieved via direct sprouting, grafting or budding. Examples include fruits (*Malus, Prunus, Morus*), forest trees (*Fraxinus, Populus*), nut trees (*Juglans*) and ornamental trees and shrubs (see references in Towill and Ellis 2008; Green and Grout 2010). For herbaceous plants, only a few studies are available, mostly on garlic (Towill and Ellis 2008).

In herbaceous plants, shoot tips for cryopreservation are usually isolated from actively growing in vitro plants. A common pre-conditioning measure is to cold-acclimate in vitro plants of temperate origin to increase their tolerance to freezing and to improve cryopreservation results. Cold acclimation leads to biochemical changes and can be induced either at constant low, non-freezing temperatures or using an alternating temperature regime (Keller 2005; Senula et al. 2007; Hincha and Zuther 2014). Typical plant responses to cold stress conditions are increased antioxidant enzyme activities and increased soluble sugar content (Nievola et al. 2017). Cold acclimation also directly affects the cell membrane because it increases the level of phospholipids such as phosphatidylcholine and phosphatidyl-ethanolamine. These improve membrane stability, fluidity and permeability, and thus freezing tolerance (Takahashi et al. 2016). More than 1000 cold-induced genes have been identified, and the exposure of plants to low temperatures triggers a highly complex regulatory programme (Thomashow 2010).

This study was designed to develop a protocol for the cryopreservation of *D. austriacum.* Two types of starting materials were used for cryopreservation: shoot tips from in vitro*-*grown plantlets and axillary buds from winter shoots of field plants. As a pre-requisite, we established micropropagation protocols including initiation from seeds and rooting of in vitro plantlets.

## Material and methods

### Initiation of in vitro plant material

Seeds were collected from the living plant collection at the Botanical Garden of the University of Vienna in 2013 (Hundsheimer Berge population) and stored at room temperature (18–30 °C) until culture initiation in October 2014.

Seeds (87 in total in two set-ups) were washed in tap water for 30 min on a stirrer and disinfected in nylon mesh bags in 70% ethanol for 3 min followed by 2.8% NaOCl (Neuber’s Enkel, Vienna, Austria) for 40 min. Then they were rinsed in sterilised water three times and cultured on ½ MS medium (Murashige and Skoog 1962) with 3% sucrose and 0.2% gelrite in individual test tubes to prevent cross contamination. Visibly uncontaminated seeds after 4–5 days were nicked with a scalpel on the pointy tip (microphyle) under a laminar hood and transferred back to the tube. Seedlings were then used for micropropagation trials.

Cultures were maintained at 25 °C ± 2 °C under 45 µmol/m^2^/s light with a 16 h photoperiod provided by fluorescent lamps (Sylvania Grolux T8 58 W) in a growth chamber.

### Micropropagation

In a preliminary trial, full and ½ MS medium with various concentrations of benzyl adenine (BAP) (0, 0.5, 1, 2.5, 4 µM) were tested for shoot propagation using single shoots or clusters of shoots. Ultimately, ½ MS medium (½ macro, full micro and vitamins) supplemented with 1 μM BAP was best suited and selected as the basic medium. The medium contained 100 mg/l myoinositol, 3% household-grade sugar and 0.2% gelrite (Duchefa, the Netherlands). The pH was set to 5.8 and all culture media were autoclaved for 20 min at 121 °C. A mix of single shoots and clusters of shoots were cultured on 40 ml medium in glass jars with a volume of 200 ml (Hipp, Gmunden, Austria), closed with Magenta B-caps (Sigma-Aldrich, US). The established material was bulk propagated under an alternating temperature regime of 22/8 °C and a 16 h photoperiod in a growth cabinet and then used for rooting and cold storage experiments. Subculturing of single shoots and shoot clusters took place every 5 to 8 weeks.

For cryopreservation, plants were propagated at alternating temperatures under three different growth regimes (1) Glass jars with non-ventilated lids as described above (2) glass jars with ventilated Magenta caps (hole size 8 mm, adhesive microfiltration discs, Sigma-Aldrich, US) that allow gas exchange in a regular culture environment and (3) ventilated glass jars in a CO_2_-enriched environment. CO_2_ was supplied from a freshly prepared bicarbonate/carbonate buffer mixture consisting of one part 3 M K_2_CO_3_ solution and three parts 3 M KHCO_3_ solution. The jars were placed inside a glass enclosure (upside-down empty fish tank) together with the buffer mixture. The buffer mixture was placed on a magnetic stirrer and renewed weekly.

### Rooting

Non-cryopreserved shoots (~ 15 mm long) were subjected to various rooting treatments: ¼ MS, ½ MS and liquid ½ MS with rockwool support containing 1 µM indole-3-butyric acid (IBA) for 4 weeks. The media contained 100 mg/l myoinositol and 3% household-grade sugar as before. For rooting experiments, the gelrite concentration was increased from 0.2 to 0.3% because this worked better in preliminary tests. After 4 weeks the plantlets were transferred to the same medium but without growth regulators for another 6 weeks. For comparison, a control treatment was carried out with continuous culture on the shoot propagation medium with 1 µM BAP for 10 weeks. Nine glass jars with four shoots each were set up per treatment.

Subsequently, an up-scaled rooting trial with 240 shoots was conducted using ½ MS with 1 µM BAP, culturing five shoots per glass jar for 10 weeks.

### Cold storage

Cultures that were not used for trials were placed into cold storage. Shoots in glass jars on propagation medium were sealed with Parafilm® and kept at alternating temperatures for about 10 days before being moved to 10 ± 2 °C for cold storage under a 16 h photoperiod.

### Cryopreservation of in vitro material

The cryopreservation process was based on protocols of mint (Senula et al. 2007) and garlic (Keller 2005). Shoot tips (2 mm) with two leaf primordia were isolated from in vitro shoots derived from the three different growing regimes described under micropropagation.

The excised shoot tips (up to 30 pieces per Petri dish) were precultured overnight (16–19 h) in 2 ml liquid full MS medium with 0.3 M sucrose at 25 °C in small Petri dishes (60 × 15 mm) lined with sterile filter paper in the light. The next morning, explants were transferred to 2 ml loading solution (2 M glycerol + 0.4 M sucrose) for 20 min. This and the following steps were carried out at room temperature. Next, the explants were incubated in 3 ml PVS3 (50% w/v sucrose + 50% w/v glycerol) (Nishizawa et al. 1993). After a 2-h incubation period, explants were transferred into 4 µL droplets of PVS3 placed on strips of sterile aluminium foil. Two of these strips with 5 droplets each and explants were placed in cryovials. The closed cryovials were plunged into liquid nitrogen (LN) in a Dewar transporting container (model 26B, 1L, from Roth, Karlsruhe, Germany). The explants remained in cryostorage for a minimum of 1 h.

Upon removal from LN storage the cryovials were rewarmed in a warm water bath at 40 °C for approximately 30 s. The foil strips were then removed and the shoot tips incubated in 6 ml washing solution (1.2 M sucrose) for 20 min at room temperature. From the washing solution the explants were transferred to a semi-solid regeneration medium (full-strength MS medium supplemented with 0.5 µM BAP, 3% sucrose) in small Petri dishes. All solutions were prepared with liquid full MS, pH adjusted to 5.8 and autoclaved.

Ten explants were incubated per Petri dish (one replicate) at 25 °C in the dark. After approximately 1 week the cultures were gradually moved to light.

For those plants coming from the non-ventilated treatment, the effect of incubation temperature was additionally tested by keeping the explants over ice during the 2-h treatment.

The final evaluation was conducted 2 months after cryopreservation. Survival was based on explants showing any growth, whereas for regeneration, explants were only counted when vigorous shoots were produced. We used 160–260 shoot tips per treatment and 500 shoot tips for the ice treatment. Statistical comparison between the in vitro treatments was conducted using the Kruskal Wallis Test in Statgraphics18. Where required, post-hoc tests were undertaken using Bonferroni intervals test. P < 0.05 was deemed significant.

### Cryopreservation of field material

**Fig. 1:**
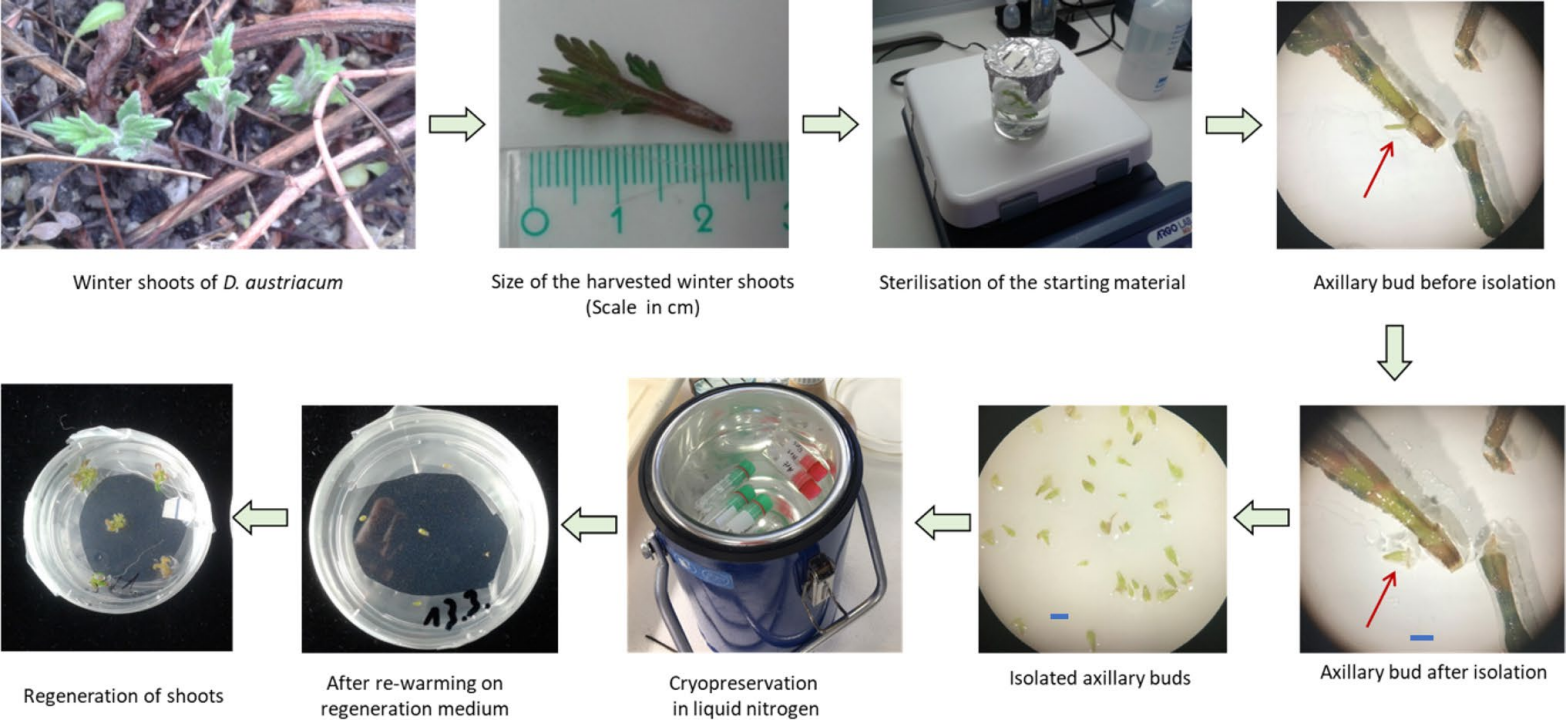
Cryopreservation of field material. Scale bars 1 mm

Winter sprouts measuring 20–30 mm (Fig. 1) were collected from *D. austriacum* plants growing in the garden beds of the University of Vienna, Spittelau. Materials were collected at 6-week intervals during winter time: 18 December 2018, 30 January and 12 March 2019. Leaf blades were removed and shoots briefly washed in tap water and then surface sterilised in 70% ethanol for 3 min and in 1.68% NaOCl for 20 min. Afterwards, the stems were briefly rinsed and then thoroughly washed with sterile water three times (5–5–10 min). Axillary buds (approx. 1 mm in length) were isolated and used in cryopreservation experiments (Fig. 1).

Four different vitrification protocols were followed: (1) vitrification protocol over ice as described under in vitro material (2) elimination of overnight preculture (3) elimination of overnight preculture and loading solution step and (4) elimination of all vitrification steps but direct transfer of buds to PVS3 droplets on foil strip. For initial tests in December, only 16 shoot tips and protocol 1 was followed. In January and March, 50 buds were used for protocol 1, and 30 buds for protocols 2, 3 and 4.

The day length was available on https://www.sunrise-and-sunset.com and temperature field data was retrieved from https://at.wetter.com/wetter_aktuell/rueckblick/oesterreich/wien/ATAT10678.html?sid=11034&timeframe=1y website for the location ‘Inner City’.

### Analysis of ploidy

Shoots regenerated after cryopreservation from in vitro material (23 samples) were evaluated for their ploidy levels and results were interpreted by comparison with plants from the living plant collection garden beds (two different populations: Wiener Wald and Hundsheimer Berge, one plant each).

For flow cytometric measurements, about 25 mg of plant material from every sample was co-chopped (Galbraith et al. 1983) with plant material from the standard organism *Solanum pseudocapsicum* (Temsch et al. 2010) in 1.1 ml isolation buffer (pH ~ 1.5, Otto et al. 1981) using a razor blade. The suspension was filtered through a 30 micron nylon mesh. 50 µL RNase A stock solution (Sigma-Aldrich, 3 mg/ml A. dest.) were added and then incubated in a 37 °C water bath for 30 min. After RNA digestion, 4 ml staining buffer (Otto et al. 1981), which contains propidium iodide (BioChemica, pH ~ 9.5), were added and the samples were incubated in the refrigerator for 60 min before the flow cytometric measurement. The samples were measured with a Partec CyFlow ML flow cytometer (Partec, Muenster, Germany) equipped with a 532 nm and 100 mW diode pumped laser (Cobolt Samba, Cobolt AB, Solna, Sweden).

A total of 3333 particles were measured within one run of the instrument. One individual per preparation was measured and the results of three runs per preparation were averaged.

The results were calculated according to the formula: 1C-value of the object = (mean fluorescence intensity of the object’s G1 nuclei population/mean fluorescence intensity of the standard’s G1 nuclei population) * 1C-value of the standard organism.

## Results

### Initiation of in vitro plant material

Of the total 87 seeds disinfected, 87% were free of bacterial and fungal contamination after 4–5 days and were nicked. Seeds typically germinated within a week after nicking. While 84% of the nicked seeds germinated, only 66% reached the seedling stage, showing roots and shoots. The remaining germinated seeds either produced only cotyledons or very small plantlets that did not develop further. Nicking facilitated germination. The 87 cultured seeds yielded a total of 50 seedlings (= 57%), which then underwent micropropagation.

### Micropropagation

Preliminary trials showed that ½ MS containing 1 µM BAP was best suited as micropropagation medium. Plants were acclimated for the cryopreservation experiments under the alternating temperature regime, where they grew vigorously and developed shoot clusters, independent of the ventilation treatment. *D. austriacum* also readily produced new shoots via direct and indirect organogenesis at the base of the stems and rarely also on the leaves (here only direct organogenesis).

Plantlets propagated in jars with regular Magenta B-caps showed signs of hyperhydricity. Passive ventilation by using caps with filters had a visible positive effect on plant quality (less glassy appearance). The positive effect was particularly pronounced in plantlets with CO_2_-supply: their colour and overall appearance resembled field-grown plants more closely.

### Rooting

The evaluation of the 2-step rooting procedure after four and 10 weeks is presented in Table 1. After 10 weeks ½ MS + 1 µM IBA treatment yielded 75% rooted explants. Keeping the plants on BAP medium up to 10-week period, however, yielded 94.4% rooted shoots.

**Table 1:** Root formation of the three IBA 2-step-rooting treatments and the control on 1 µM BAP

Medium	Growth regulator first 4 wks	% Explants with roots after 4 wks	Growth regulator subsequent 6 wks	% Explants with roots after 10 wks
¼ MS	1 µM IBA	11.1	0	47.2
½ MS	1 µM IBA	2.8	0	75.0
½ MS liquid/rock wool	1 µM IBA	19.4	0	25.0
½ MS	1 µM BAP	–	1 µM BAP	94.4

First evaluation took place after 4 weeks (wks) during subculturing, the final after 10 weeks. (36 explants per treatment)

The success of the ½ MS + 1 µM BAP treatment was confirmed in the large-scale experiment which yielded 90.4% rooting in a sample of 240 shoots.

### Cold storage

While cultures at alternating temperatures needed to be subcultured at regular intervals with a maximum of 12 weeks to prevent necrosis, cultures in cold storage remained viable for at least 6 months. Although some shoot clusters became necrotic by then, a sufficient number of shoots always survived to further multiply the cultures.

### Cryopreservation of in vitro material

The growing conditions had an influence on the plant material’s texture. Plants grown under enriched CO_2_ conditions enabled an easier and faster isolation of shoot tips for cryopreservation. The tissue was less likely to burst or tear upon cutting compared to swollen hyperhydric tissue.

While improved ventilation alone or in combination with CO_2_ had a positive effect on the quality of micropropagated plants, it did not have a significant effect on the plants’ cryotolerance (Table 2). Regeneration increased from 16 to 24 and 27% with increased ventilation, but statistically there was no significant difference. However, cooling the explants during PVS3 incubation over ice resulted in significantly higher survival and regeneration rates than incubation at room temperature using non-ventilated material.

**Table 2:** Survival and regeneration percentages of cryopreserved shoot tips using four different pre-treatments of stock cultures or explants

Treatment	No. of Petri dish	Survival (%)	Regeneration (%)
Non-ventilated lids	21	23.3 ± 20.3^a^	16.2 ± 18.0^b^
Ventilated lids	26	31.7 ± 22.3^a^	24.2 ± 22.0^a,b^
Ventilated lids and CO_2_-enriched	16	30.6 ± 18.8^a^	26.9 ± 16.2^a,b^
Non-ventilated lids + explants on PVS over ice	50	37.6 ± 22.6^a^	34.2 ± 23.0^a^

Data represents mean ± SD of 10 shoot tips/Petri dish. Data with same alphabet in a column are not significantly different as per Kruskal–Wallis Test at 95% confidence level

During regeneration, shoots sometimes arose from the shoot tip meristem but also through direct organogenesis, with adventitious shoots arising from leaves or the base of the explants without callus formation. The success of the cryopreservation varied strongly from one Petri dish to another as well as between different experimental days.

### Cryopreservation of field material

The minimum and maximum temperatures that the plants were exposed to 10 days before the harvest of winter shoots were − 1.4/10.5 °C in December, − 5.9/6.4 °C in January and 3.6/20.4 °C in March. The day length was 8 h 19 min on 18 December, 9 h 22 min on 30 January and 11 h 38 min on 12 March.

**Fig. 2:**
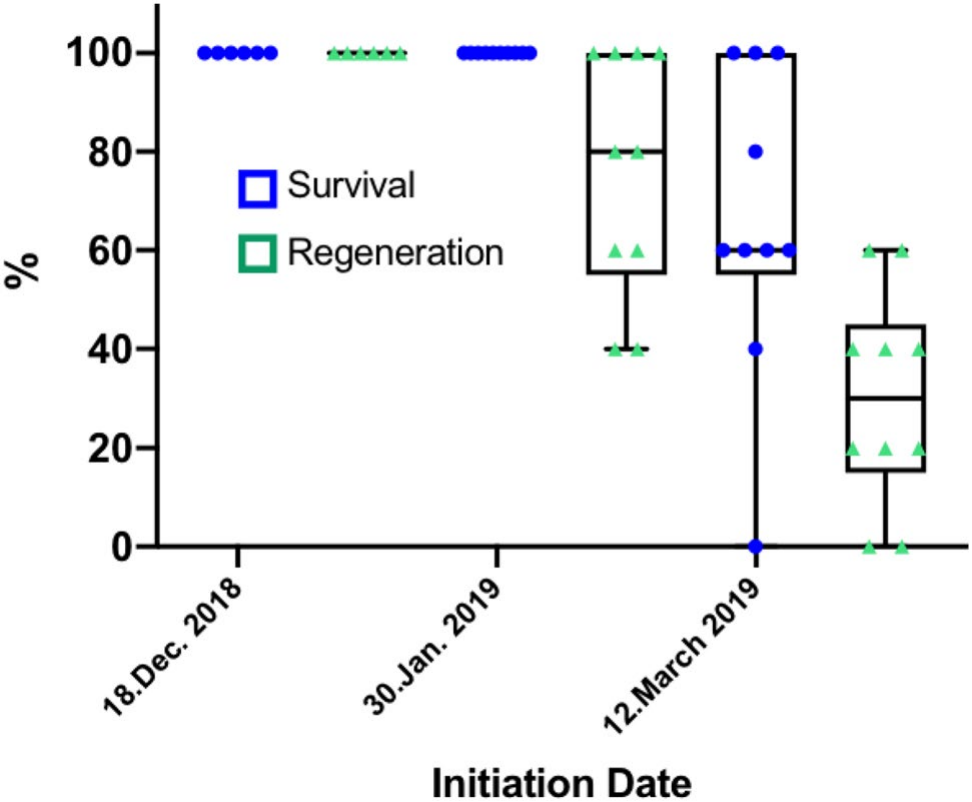
Box and Whisker Plot of survival and regeneration percentages of the cryopreserved field materials initiated in different months using the full protocol. The boxes display the dataset based on sample median and the first and third quartiles, whiskers represent the min and max values, the triangles and circles the mean per Petri dish

Winter acclimated buds showed up to 100% regeneration, which was three times higher than for in vitro shoot tips and they also developed faster (Table 3). The simple sterilisation protocol worked well and no contamination occurred in the cultures during regeneration. Growth was visible within 2–3 days, and shoots were moved to low light conditions. During regeneration, direct and indirect organogenesis was observed. Best results (100% survival and regeneration) were achieved in December (early winter) (Fig. 2). Season was an important factor as regeneration dropped to 76% in January and to 30% towards spring (March). An ANOVA was not feasible due to a lack of variance in some data sets and we decided to provide a graphical analysis exclusively. The box and whisker plots display the dataset based on sample median, the first and third quartiles and the minimum and the maximum.

**Table 3:** Survival and regeneration of cryopreserved shoot tips using the full and simplified protocols

Treatment	18 December 2018		30 January 2019			12 March 2019		
	Survival %	Regeneration %	Survival %		Regeneration %	Survival %		Regeneration %
1. Full protocol	100 ± 0	100 ± 0	100 ± 0		76.0 ± 24.6	66.0 ± 31.3		30.0 ± 21.6
2. LS & PVS3	–	–	93.3 ± 10.3		33.3 ± 27.3	30.0 ± 27.6		20.0 ± 17.9
3. Only PVS3	–	–	86.7 ± 10.3		26.6 ± 10.3	13.3 ± 16.3		6.7 ± 10.3
4. Into PVS droplets	–	–	6.7 ± 10.3		3.3 ± 8.2	–		–

Data represents mean ± SD of 10 shoot tips/Petri dish

*LS* loading solution, *PVS3* plant vitrification solution 3

**Fig. 3:**
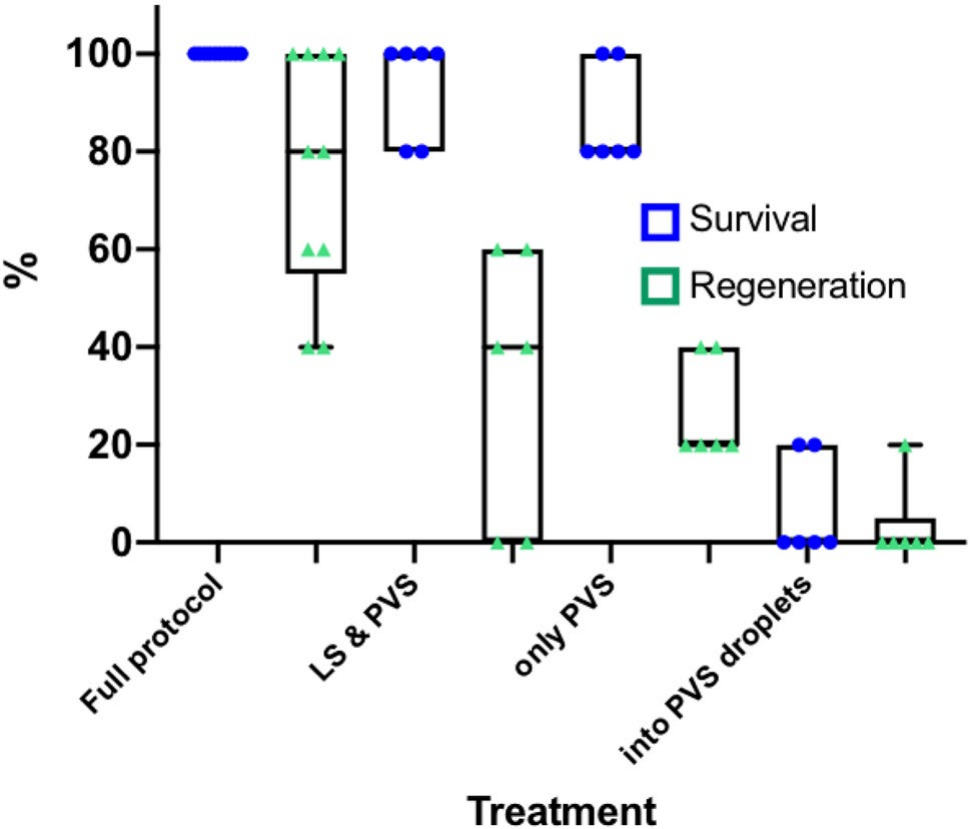
Box and Whisker Plot of survival and regeneration percentages of the cryopreserved field material initiated on the 30th of January 2019. The boxes display the dataset based on sample median and the first and third quartiles, whiskers represent the min and max values, the triangles and circles the mean per Petri dish. *LS* loading solution, *PVS* plant vitrification solution

Eliminating individual steps in the vitrification process strongly influenced the results (Table 3). Leaving out only the overnight culture and/or the loading solution in January still showed a high survival of tissue, but regeneration decreased strongly (Fig. 3). When buds were directly placed into the PVS droplets, only two of the 30 explants survived and one regenerated into a plantlet. This treatment was thus not repeated in March. Success in March was generally low, and was pushed even lower when a reduced vitrification protocol was applied.

### Analysis of ploidy

The 1C-values of the different materials (25 samples in total) ranged from 0.7191 pg to 0.7537 pg with a mean 1C-value of 0.7376 pg (STDEV 0.0082). The mean 1C-value of the 23 cryopreserved samples of in vitro origin was 0.7391 pg (STDEV 0.0071). The ratio max/min 1C was 1.036. The 1C of the field plants for Wiener Wald was 0.7311 and 0.7191 for the Hundsheimer Berge. Thus there was no indication of polyploidy induction by cryopreservation.

The CV% values of the peaks in the histograms usually ranged from 2 to 3% but in a few exceptions reached up to 5%. The CV% (CV% = standard deviation/mean*100) provides information about sample quality. A CV% ≤ 3% is desirable, but in plants containing essential oils such as *Dracocephalum*, values ≤ 5% are acceptable.

## Discussion

This is a rare report on a dicotyledonous herbaceous plant being cryopreserved using winter-acclimated buds from field material. Winter shoots were first sterilised, then axillary buds isolated and cryopreserved using a vitrification protocol. The regeneration of plants after rewarming was undertaken in vitro. The use of naturally cold-acclimated field material proved to be more successful for the cryopreservation of *D. austriacum* than using shoot tips from cold-acclimated in vitro plants.

Both approaches depended on a reliable micropropagation protocol, which had to be established first. Limited starting material was available for the establishment of laboratory experiments because of the endangered nature of the species, but seeds from the living collection could be spared. Preliminary trials on the germination of seeds in vitro were unsuccessful; mechanical scarification by nicking the seed coat was necessary. Another species of *Dracocephalum*, *D. kotschyi*, is also known to have dormant seeds and to require pre-treatment involving stratification or scarification (Moradi and Otroshy 2012a). The micropropagation of shoots was quickly accomplished on medium with cytokinin BAP (1 µM). Rooting in *D. austriacum* was highest after prolonged culture on the same medium as used for propagation. In *D. kotschyi*, rooting was also reported on BAP medium in combination with naphthalene acetic acid (NAA) (Moradi and Otroshy 2012b). Few reports are available on plants preferring cytokinin-based medium for rooting, *e.g.* the sedge *Gahnia radula* (Kodym et al. 2014).

In vitro plants proliferated well despite being affected by hyperhydricity. Nevertheless, negative effects on cryopreservation were anticipated. As increasing the ventilation is considered one way to combat hyperhydricity (Bhatia et al. 2015), ventilated lids alone or in combination with a CO_2_-enriched environment were tested. We recorded a positive effect on plant quality using ventilated lids and even more so in plantlets grown with increased CO_2_ supply. However, using plantlets of better quality did not significantly improve regeneration after cryopreservation. The significance between the various treatments may have been diminished by the high degree of variance in the results from day to day and from Petri dish to Petri dish. On the other hand, the in vitro material benefited from cooling the shoot tips during PVS3 incubation by keeping them over ice. It was demonstrated in mint, that glycerol and other cryoprotectants were less damaging when shoot tips were exposed at 0 °C compared to exposures at 22 °C (Volk et al. 2006). Incubation at zero degrees is thus commonly used in vitrification protocols (Chen et al. 2011; Kaczmarczyk et al 2012). In a more recent study, regeneration rates could be improved by lowering the sucrose concentration of the washing solution. In potato, 0.6 M was better suited for most genotypes at the International Potato Center (CIP) and has now replaced the commonly used 1.2 M (Vollmer et al. 2019).

The vitrification protocol with PVS3 over ice was well suited for *D. austriacum* field material, as documented by the 100% regeneration. Using field material had the major advantages of shortening the preparation time and reducing preparatory work. Several axillary buds could be fairly quickly harvested from one shoot applying the correct isolation technique, without harming or threatening the survival of the mother plant. In view of endangered species, where seed production may be low, or species are cross pollinated, this method represents a quick possibility to conserve the genetic diversity of the remnant population. Established in vitro cultures are often preferred as starting material for cryopreservation over field material because of the fear of introducing contamination by microbes (Nukari et al. 2009). However, this potential disadvantage of field material never materialized: clean cultures could be established using the described sterilisation method in combination with very small explants that were protected by leaf bases with 100% success. The material from field plants not only yielded higher regeneration rates but also had shorter regeneration times, even though the explant size of the axillary buds was much smaller than that of the in vitro shoot tips. The positive results of winter season buds indicate they have potential not only for cryopreservation, but also for tissue culture, *i.e.* in providing relatively clean explant material in establishing contamination-free cultures. The transfer of in vivo plant materials to in vitro conditions is often the most difficult step in tissue culture procedures and may preclude tissue culture altogether. Winter buds may therefore provide a means of over-coming this obstacle.

The only other report on cryopreserving winter buds in an herbaceous dicotyledonous plant has been on peony (Seo et al. 2007). Dormant shoot tips were harvested from winter buds after surface sterilisation and desiccated under a laminar air-flow, before being plunged into LN. The authors likewise did not report on any problems with contamination. Survival after cryopreservation depended on the collection date and the duration of the desiccation process.

Dormant buds from woody plants such as trees or berry fruit for cryopreservation are generally harvested mid-winter, although the optimal time of harvest can vary from year to year depending on the weather conditions (Towill and Ellis 2008). The time of collection of field material is a crucial factor, and the present study clearly demonstrates the importance of determining the narrow time window of deep acclimation. The optimal cold-hardiness in *D. austriacum* in Vienna was in December with temperatures below freezing briefly before harvest and a daylength of around 8 h. Temperatures below freezing have a beneficial effect on cryotolerance while the decrease of survival and regeneration over time could be linked with the increase in photoperiod: short day conditions are known to strongly favour cold-acclimation. In peony, the highest regrowth (66–74%) was obtained between late November and late February and dropped to less than 1% in late March (Seo et al. 2007). In Finland, for example, the regeneration in aspen was only 9.8% in September but increased to 75% from October to February (Aronen and Ryynanen 2014).

Protocols for cryopreservation vary between species: some species can be directly frozen, whereas for others the excised buds must first be treated with a series of cryoprotectants and then frozen. Based on the high regeneration rate in *D. austriacum* in December, we proposed the hypothesis that the natural cold acclimation is so advanced that the vitrification protocol can be simplified. Omitting one or more steps, however, led to a drastic decrease in regeneration. A similar observation was made in peony, where non-desiccated shoot tips showed poor recovery (Seo et al. 2007).

Since shoot formation often occurred from the base of explants via organogenesis rather than the apical meristem, we assessed the genetic stability of cryopreserved *D. austriacum *in vitro plants using flow cytometry. No changes of ploidy level were detected. These results are consistent with multiple previous reports that cryopreservation is generally considered a safe method for long-term germplasm conservation (e.g. Adu-Gyamfi et al. 2010; Carmona-Martín et al. 2018).

This study clearly shows that winter shoots from herbaceous plants can be an excellent source for cryopreservation. This calls for exploring this technique for other temperate, herbaceous perennials such as strawberries or subshrubs.
